# Development and validation of the pulmonary tuberculosis scale of the system of Quality of Life Instruments for Chronic Diseases (QLICD-PT)

**DOI:** 10.1186/s12955-018-0960-5

**Published:** 2018-07-11

**Authors:** Yanchun Sun, Zheng Yang, Chonghua Wan, Chuanzhi Xu, Liuping Chen, Lin Xu, Xiaoqing Zhang, Fei Yan

**Affiliations:** 1 Department of Social Medicine, School of Public Health, National Key Laboratory of Health Technology Assessment (National Health and Family Planning Commission), Collaborative Innovation Center of Social Risks Governance in Health, Fudan University, Shanghai, 200032 China; 2 School of Public Health, Guangdong Medical University, Dongguan, 523808 China; 3 School of Humanities and Management, Research Center for Quality of Life and Applied Psychology, Guangdong Medical University, Dongguan, 523808 China; 4 School of Public Health, Kunming Medical University, Kunming, 650500 China; 5 Yunnnan Center for Disease Control and Prevention, Kunming, 650022 China

**Keywords:** Quality of life, Disease-specific instrument, QLICD-GM, Pulmonary tuberculosis

## Abstract

**Background:**

Generic assessments are less responsive to subtle changes due to specific diseases, making it challenging to fully understand the impact of pulmonary tuberculosis (TB) on patient’s quality of life (QOL).

**Methods:**

We applied programmed decision procedures and theories on instrument development to develop the scale. Two hundred patients with pulmonary TB participated in measuring QOL three times before and after treatments. We assessed the validity, reliability, and responsiveness of QLICD-PT using correlation analysis, factor analysis, multi-trait scaling analysis, randomized block analyses of variance with Least Significant Difference post-hoc tests.

**Results:**

We composed QLICD-PT with 3 domains (28 items) for general QOL and 1 pulmonary TB specific domain (12 items). Correlation and factor analysis confirmed good structure validity and criterion-related validity when using Chinese version of the Medical Outcomes Short-Form Health Survey (SF-36) as a criterion. The internal consistency of α values were higher than 0.70. The score changes after treatment were of statistical significance for the overall scale, physical domain and specific domain with effect size ranging from 0.32 to 0.72. No floor effects but small ceiling effects were observed at domain level.

**Conclusions:**

As the first pulmonary TB-specific QOL scale developed by a module approach in Chinese, QLICD-PT has an acceptable degree of validity, reliability and responsiveness, and can be used to measure the life quality of PT patients specifically and sufficiently.

## Background

Pulmonary Tuberculosis (TB) is a chronic pulmonary infection caused by *Mycobacterium tuberculosis*. As a major global public health challenge, TB remained one of the top 10 causes of deaths worldwide, leading to more deaths than HIV/AIDS did [1]. According to World Health Organization, around 9.6 million people were diagnosed with TB in 2014, 1.2 million died from the disease [2]. With almost one million new cases in 2015, approximately 10% of the global incident cases, TB continues to be a major public health problem in China, and making China the third among the high TB burden countries [3].

Compared with the general population, patients with TB reported more deficits in their physical and mental well-being. TB patients are facing various formats of social rejections and isolations because TB has been stigmatized as a source of infection for the healthy individuals [4–6], which may lead to work absenteeism, and in turn, substantial amounts of loss of productivity, and reduced monthly income. Stigmatization and negative emotions resulting from the illness could result in a long-term impairment of patient’s psychosocial well-being [7]. Emerging evidence also suggest that psychosocial burden amongst TB patients, after microbiological cure, may have a greater impact on health-related quality of life (HRQOL) than clinical symptoms [5, 8], and QOL of TB patients has been substantially compromised [7–9].

The term quality of life (QOL) and HRQOL have been created to pivot a collection of health outcome research over the past decades. The term HRQOL is often used to indicate QOL from the perspective of health care or medical services people experience [10], hence, in this study, the term of QOL and HRQOL are interchangeable. QOL instruments are usually classified as being either generic or disease-specific. Generic measures can be used in almost any population, irrespective of the underlying condition or disorder. Since generic measures apply to a wide variety of populations, they allow for broad comparisons of relative impact of various diseases or interventions on QOL [11, 12]. However, generic assessments are less responsive to subtle changes due to specific diseases or in specific population. The disease-specific instruments have the advantage for assessing domains relevant to specific diseases and sensitive to capture small changes [11, 12]. However, to the best of our knowledge, one study has been published to assess QOL for patients with TB [13], making it challenging to fully understand the impact of TB on patient’s QOL. Therefore, it is urgent to develop a tuberculosis-specific QOL scale under the Chinese culture context.

The Chinese QOL instruments, i.e. Quality of Life Instruments for Chronic Diseases (QLICD), were developed, including both a generic module (QLICD-GM) [14], and various modified modules for specific diseases. Instruments have been developed and validated for coronary heart disease (QLICD-CHD) [15], irritable bowel syndrome (QLICD-IBS) [16] and for hypertension (QLICD-HY) [17] but not pulmonary TB, the leading causes of mortality and morbidity of infectious diseases among Chinese. Therefore, we made effort to make the missing piece of the puzzle and develop a set of QLICD, specifically for the Pulmonary TB (QLICD-PT). The aim of this paper was to describe the development and validation processes of QLICD-PT.

## Methods

### Development of the QLICD-PT

#### General principles and steps of developing QLICD-PT

**Fig. 1:**
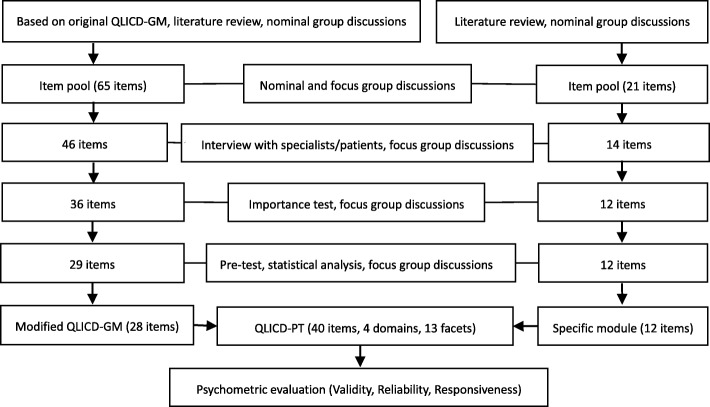
Steps towards development and validation procedure of QLICD-PT

In principle, our effort to develop QLICD-PT followed the general steps described in detail elsewhere for QLICD-GM [14]. In brief, the QLICD-PT was created from two sub-modules, i.e., modified QLICD-GM (very left column of the Fig. 1) and newly created pulmonary TB specific module (very right column of the Fig. 1). We approached both modified QLICD-GM and pulmonary TB specific module by two mutually independent group efforts. The nominal group consisted of 16 individuals including six physicians, two nurses, one medical educator, and seven teachers/researchers (two in QOL/medical statistics, one in epidemiology, two in sociology, two in psychology), and were created to make suggestions about what items should be included. The focus group with 10 experts including four physicians, one medical educator, and five teachers/ researchers (two in QOL/medical statistics, one in epidemiology, one in sociology, one in psychology) were formed to use programmed decision method to present the conceptual framework and select items proposed by nominal group. Overall, the nominal group was responsible for item presentation, whereas the focus group dealt with item selection and organization. During the process of item selection, we applied not only qualitative analysis such as group discussion, in-depth interviews, pilot tests and pretests, but also quantitative statistical methods of variation analysis, correlation analysis, factor analysis and cluster analysis procedures.

#### Modifying QLICD-GM

Slight modification was made to simplify the original version of QLICD-GM [14]. For example, sexual function was an independent facet in original QLICD-GM but included as part of the physiological functions in modified QLICD-GM. Modified QLICD-GM consists of 28 items, classified into three domains and nine facets. Physical domain (PHD) includes 9 items (coded GPH1-GPH9) grouped into three facets: Basic Physiological Functions (BPF), Energy and Discomfort (EAD), and Independence (IND). Psychological domain (PSD) contains of 11 items (coded GPS1-GPS11), divided into three facets: Cognition (COG), Will and Personality (WIP), and Emotion (EMO). Social domain (SOD) comprises 8 items (coded GSO1-GSO8), categorized into three facets: Interpersonal Communication (INC), Social Support and Security (SSS), and Social Role (SOR) (Table 1).

**Table 1:** The construct and scoring method of the quality of life instrument QLICD-PT

Domains/facets	Number of items	Range scores	Scoring method	
			Raw score	Standardized score
Physical domain (PHD)	9	9–45	BPF + IND + EAD	(RS-9) × 100/36
Basic physiologic functions (BPF)	4	4–20	GPH1 + GPH2 + GPH3 + GPH4	(RS-4) × 100/16
Independence (IND)	3	3–15	GPH6 + GPH7 + GPH8	(RS-3) × 100/12
Energy and discomfort (EAD)	2	2–10	GPH5 + GPH9	(RS-2) × 100/8
Psychological domain (PSD)	11	11–55	COG+EMO + WIP	(RS-11) × 100/44
Cognition (COG)	2	2–10	GPS1 + GPS2+	(RS-2) × 100/8
Emotion (EMO)	7	7–35	GPS3 + GPS4 + GPS5 + GPS6GPS7+ GPS8 + GPS9	(RS-7) × 100/28
Will and personality (WIP)	2	2–10	GPS10+ GPS11	(RS-2) × 100/8
Social domain (SOD)	8	8–40	INC + SSS + SOR	(RS-8) × 100/32
Interpersonal communication (INC)	3	3–15	GSO1 + GSO2 + GSO3	(RS-3) × 100/12
Social support and security (SSS)	3	3–15	GSO4 + GSO5 + GSO6	(RS-3) × 100/12
Social role (SOR)	2	2–10	GSO7 + GSO8	(RS-2) × 100/8
Sub-total (QLICD-GM)	28	28–140	PHD + PSD + SOD	(RS-28) × 100/112
TB Specific domain (SPD)	12	12–60	RES + COS + DSE + SPM	(RS-12) × 100/48
Respiratory symptom (RES)	6	6–30	PT1 + PT2 + PT3 + PT4 + PT5 + PT6	(RS-6) × 100/24
Constitutional symptom (COS)	3	3–15	PT7 + PT8 + PT12	(RS-3) × 100/12
Drug side-effects (DSE)	1	1–5	PT9	(RS-1) × 100/4
Special mention (SPM)	2	2–10	PT10 + PT11	(RS-2) × 100/8
Total (TOT)	40	40–200	PHD + PSD + SOD+SPD	(RS-40) × 100/160

*RS* raw score, *SS* standardized score

#### Creating pulmonary TB specific module

Using similar procedure described above, we selected 21 items as the item pool of pulmonary TB specific module based on literature reviews, nominal/focus group discussion and patient interviews. A total of 4 facets with 12 items (coded PT1-PT12) made the way to the final module, covering Respiratory Symptom (RES), Constitutional Symptom (COS), Drug Side-effects (DSE) and Special Mention (SPM) to pulmonary TB (see Table 1).

### Validation of the QLICD-PT

#### Data collection and scoring

The QLICD-PT, combining both modified QLICD-GM and pulmonary TB specific facets, was used to evaluate patients with pulmonary TB in a field survey for assessing the psychometric properties. The survey was carried out in ten Disease Control and Prevention Centers selected in Yunnan Province, China. The study population was limited to patients with pulmonary TB who were able to read and understand the questionnaire. The participating investigators included doctors, nurses, and medical postgraduates. The investigators explained the purpose and the scale to the patients and obtained informed consent from patients who agreed to participate in the study. The study protocol and informed consent form were approved by the Institutional Review Board of the investigators’ institutions. Each respondent (*n* = 200) completed the questionnaire before receiving treatment as the 1st wave of assessment. After 2 months of treatment, respondents (*n* = 198) participated in the 2nd wave of assessment, and after 6 months of treatment, a total of 175 respondents participated in the 3rd wave of assessment to evaluate responsiveness.

Due to lack of an agreed-upon gold standard for assessing QOL of pulmonary TB, and convergent and discriminant validity of QLICD-PT, we used the Chinese version of the Medical Outcomes Study 36-Item Short-Form Health Survey (SF-36) [18], one of the commonly used generic QOL scales to collect data for assessing the criterion-related validity of QLICD-PT. SF-36 included eight subscales: Physical Function (PF), Role-Physical (RP), Bodily Pain (BP), General Health (GH), Vitality (VT), Social Function (SF), Role-Emotional (RE), and Mental Health (MH).

#### Analytic steps and indicators used to measure the validity

Each item of QLICD-PT is rated in a five-level Likert scoring system, namely, not at all, a little bit, somewhat, quite a bit, and very much. The positively stated items were scored from one to five, while the negatively stated items were scored from five to one. By adding together within the domain/facet item scores, we obtained the raw scores by items, facets, and domains. The overall score of the scale is the sum of all domain scores. For the purpose of comparison, all the domain scores were linearly converted into a standardized score (SS) ranging from 0 to 100 using the following equation: SS = (RS - Min) × 100/R, where RS, Min, and R represent the raw score, minimum score, and range of scores, respectively (see Table 1 for details). We assessed the validity of QLICD-PT from perspective of validity (construct validity, content validity), reliability (internal consistency), and responsiveness as recommended [13].

#### Construct validity

We calculated Pearson’s correlation coefficient between the similar domains of QLICD-PT and SF-36 to assess convergent validity, one aspect of construct validity. Multi-trait scaling analysis [19] was applied to test item convergent and discriminant validity with the following two criteria: convergent validity is supported when the item-domain/facet correlation is 0.40 or above; and discriminant validity is showed when the item-domain/facet correlation is higher than that of other domains/facets. We performed factor analysis with Varimax Rotation to examine the coincidence between components extracted from data and theoretical construct of the instrument, and confirm the construct validity.

### Content validity

The floor and ceiling effects are characterized by scores being concentrated on the lowest and highest sides of the overall distribution, respectively. If floor and ceiling effects are present, it is likely that extreme items are missing in the lower or upper end of the scale, indicating limited content validity. As a result, patients with the lowest or highest score can’t be distinguished from each other, thus reliability is reduced [20]. The floor and ceiling effects of each domain/facet were evaluated. Floor and ceiling effects were defined to be present if more than 15% of the patients reported the minimum or maximum possible score [20].

### Internal consistency (reliability)

Cronbach’s alpha coefficient is common practice in scale development to evaluate the internal consistency of reliability. A score between 0.70 and 0.95 has been suggested as evidence of adequate internal consistency [19]. To assess internal consistency, Cronbach’s alpha coefficient was calculated separately for each domain/facet.

#### Responsiveness

Responsiveness has been defined as the ability of a questionnaire to detect clinically important changes over time [20]. We measured the responsiveness by comparing the mean difference of the pre- and post- treatment assessments. Testing with randomized block analyses of variance and Least Significant Difference post-hoc tests. The standardized response mean (SRM), the measurement of effect size, was also used to proxy the responsiveness, and the values of 0.20, 0.50, and 0.80 representing small, moderate, and large effect, respectively [21] .

## Results

### Socio-demographic characteristics of the sample

The 200 patients with pulmonary TB varied in age from 15 to 79 years with a median age of 37.0 and a mean age of 39.2 ± 16.5 years old. About two thirds (68.0%) were men, and 131 (65.5%) were of Han ethnicity, including minorities of Yi, Bai, Hui, etc. The majority were currently married (133 cases, 66.5%), while 67 (33.5%) were single or previously married. Regarding of education level, 71 (35.5%) completed primary school, while 107 (53.5%) were graduates of middle school and 22 (11.0%) had a college or post-graduate degree. More than half of the study participants were farmers (*n* = 116, 58.0%), with 15 cases (7.5%) of workers, 4 (2.0%) cases of governmental officers, 65 (32.5%) unspecified cases. Government-sponsored health insurance program covered 70% of the cases (*n* = 141, 70.5%).

### Validity

Correlation analyses showed that there were strong associations between items and their own domains/facets (most correlation coefficients are higher than 0.5), but weak relationship between items across domains/facets and between domains/facets (Table 2). For example, correlation coefficients between items of GPH1-GPH9 (in bold) are higher than those across domains.

**Table 2:** Correlation coefficients r among items and domains/facets of QLICD-PT (*n* = 200)

Item	Physical domain			PHD	Psychological domain			PSD	Social domain			SOD	Specific domain				SPD
	BPF	IND	EAD		COG	EMO	WIP		INC	SSS	SOR		RES	COS	DSE	SPM	
GPH1	**0.69**	0.33	0.33	**0.57**	0.31	0.30	0.25	0.34	0.24	0.17	0.36	0.31	0.26	0.12	0.21	0.12	0.47
GPH2	**0.68**	0.25	0.23	**0.49**	0.30	0.20	0.24	0.27	0.31	0.16	0.24	0.30	0.21	0.09	0.06	0.07	0.19
GPH3	**0.56**	0.21	0.38	**0.47**	0.38	0.28	0.25	0.34	0.18	0.19	0.23	0.25	0.16	0.07	0.17	0.21	0.21
GPH4	**0.63**	0.25	0.14	**0.44**	0.26	0.23	0.25	0.28	0.38	0.21	0.21	0.33	0.13	0.12	0.05	0.08	0.15
GPH5	0.35	0.36	**0.81**	**0.60**	0.41	0.40	0.27	0.44	0.20	0.35	0.36	0.38	0.35	0.15	0.19	0.21	0.36
GPH6	0.37	**0.89**	0.28	**0.71**	0.45	0.27	0.35	0.38	0.45	0.40	0.36	0.50	0.17	0.21	0.25	0.03	0.21
GPH7	0.36	**0.85**	0.47	**0.75**	0.55	0.27	0.32	0.39	0.29	0.40	0.44	0.47	0.28	0.28	0.27	0.07	0.32
GPH8	0.32	**0.90**	0.36	**0.72**	0.45	0.30	0.40	0.40	0.40	0.37	0.39	0.48	0.12	0.17	0.24	0.02	0.17
GPH9	0.37	0.35	**0.83**	**0.61**	0.44	0.52	0.30	0.53	0.17	0.26	0.36	0.32	0.38	0.16	0.22	0.19	0.38
GPS1	0.43	0.57	0.35	0.59	**0.82**	0.31	0.49	**0.53**	0.38	0.41	0.42	0.50	0.27	0.32	0.29	0.10	0.34
GPS2	0.40	0.37	0.51	0.53	**0.84**	0.48	0.40	**0.63**	0.25	0.29	0.43	0.40	0.26	0.12	0.25	0.22	0.30
GPS3	0.25	0.13	0.16	0.22	0.16	**0.23**	0.16	**0.24**	0.27	0.10	0.14	0.21	0.00	−0.03	−0.06	−0.07	−0.03
GPS4	0.17	0.06	0.32	0.21	0.26	**0.57**	0.35	**0.54**	0.02	0.11	0.22	0.14	0.19	0.13	0.25	0.26	0.27
GPS5	0.28	0.31	0.36	0.40	0.41	**0.63**	0.36	**0.62**	0.15	0.31	0.38	0.34	0.24	0.18	0.25	0.27	0.33
GPS6	0.24	0.19	0.37	0.32	0.25	**0.69**	0.30	**0.61**	0.10	0.14	0.33	0.23	0.32	0.22	0.21	0.44	0.43
GPS7	0.28	0.26	0.45	0.40	0.36	**0.76**	0.50	**0.73**	0.27	0.33	0.39	0.41	0.29	0.23	0.27	0.26	0.37
GPS8	0.34	0.26	0.45	0.43	0.40	**0.78**	0.54	**0.76**	0.34	0.24	0.37	0.39	0.34	0.25	0.31	0.28	0.42
GPS9	0.22	0.18	0.35	0.30	0.25	**0.71**	0.46	**0.65**	0.26	0.27	0.41	0.39	0.23	0.16	0.27	0.22	0.30
GPS10	0.27	0.41	0.22	0.40	0.39	0.33	**0.75**	**0.49**	0.48	0.47	0.37	0.55	0.19	0.15	0.23	0.11	0.23
GPS11	0.31	0.19	0.29	0.32	0.41	0.58	**0.74**	**0.67**	0.13	0.26	0.38	0.31	0.26	0.08	0.21	0.14	0.27
GSO1	0.34	0.43	0.22	0.44	0.44	0.30	0.43	0.41	**0.78**	0.46	0.45	**0.70**	0.17	0.21	0.27	0.29	0.29
GSO2	0.33	0.24	0.15	0.31	0.19	0.18	0.20	0.22	**0.76**	0.25	0.25	**0.52**	0.08	0.15	0.09	−0.05	0.09
GSO3	0.31	0.24	0.13	0.30	0.16	0.21	0.27	0.25	**0.76**	0.30	0.19	**0.52**	0.07	0.12	0.11	0.08	0.12
GSO4	0.18	0.38	0.19	0.33	0.28	0.17	0.39	0.27	0.42	**0.66**	0.31	**0.59**	0.07	0.08	0.09	−0.06	0.07
GSO5	0.22	0.31	0.20	0.32	0.26	0.18	0.39	0.27	0.44	**0.78**	0.37	**0.67**	0.10	0.04	0.06	0.13	0.12
GSO6	0.20	0.25	0.35	0.33	0.33	0.33	0.25	0.37	0.13	**0.62**	0.39	**0.48**	0.23	0.22	0.20	0.28	0.32
GSO7	0.32	0.25	0.36	0.38	0.36	0.52	0.39	0.53	0.22	0.33	**0.81**	**0.55**	0.24	0.25	0.32	0.39	0.38
GSO8	0.30	0.48	0.28	0.47	0.43	0.25	0.38	0.36	0.45	0.50	**0.72**	**0.68**	0.12	0.17	0.21	−0.05	0.14
PT1	0.17	0.10	0.26	0.21	0.16	0.20	0.13	0.20	0.06	0.06	0.14	0.10	**0.72**	0.26	0.10	0.17	**0.61**
PT2	0.20	0.07	0.23	0.20	0.16	0.14	0.12	0.17	0.02	0.04	0.11	0.07	**0.73**	0.28	0.21	0.13	**0.63**
PT3	0.28	0.21	0.44	0.37	0.35	0.36	0.32	0.40	0.15	0.16	0.20	0.21	**0.82**	0.22	0.44	0.35	**0.77**
PT4	0.23	0.08	0.37	0.26	0.23	0.34	0.30	0.35	0.12	0.18	0.19	0.20	**0.75**	0.28	0.29	0.25	**0.69**
PT5	0.06	0.19	0.16	0.18	0.16	0.22	0.09	0.21	0.17	0.17	0.13	0.19	**0.54**	0.24	0.26	0.08	**0.48**
PT6	0.31	0.37	0.43	0.46	0.32	0.35	0.32	0.39	0.15	0.30	0.28	0.30	**0.68**	0.22	0.34	0.20	**0.62**
PT7	0.27	0.38	0.34	0.42	0.35	0.35	0.23	0.38	0.30	0.28	0.42	0.41	0.39	**0.80**	0.33	0.25	**0.60**
PT8	0.15	0.23	0.24	0.26	0.27	0.27	0.17	0.29	0.19	0.19	0.27	0.27	0.39	**0.85**	0.29	0.19	**0.59**
PT9	0.20	0.29	0.25	0.32	0.32	0.35	0.30	0.39	0.23	0.18	0.35	0.31	0.38	0.27	**1.00**	**0.26**	**0.54**
PT10	0.11	0.06	0.21	0.15	0.16	0.30	0.06	0.26	0.12	0.13	0.17	0.17	0.28	0.24	0.30	**0.87**	**0.55**
PT11	0.24	0.02	0.21	0.18	0.18	0.37	0.23	0.35	0.18	0.22	0.26	0.27	0.20	0.09	0.14	0.84	**0.42**
PT12	−0.20	− 0.21	− 0.34	−0.30	−0.22	− 0.21	− 0.17	−0.24	−0.14	−0.21	−0.26	−0.25	−0.21	**0.15**	−0.19	− 0.12	**− 0.16**

The numbers in bold are aimed to show strong correlations between items and their own domains/facets easily

*PHD* physical domain, *BPF* basic physiological functions, *IND* independence, *EAD* energy and discomfort, *PSD* psychological domain, *COG* cognition, *EMO* emotion, *WIP* will and personality, *SOD* social domain, *INC* interpersonal communication, *SSS* social support and security, *SOR* social role, *SPD* specific domain, *RES* respiratory symptoms, *COS* constitutional symptom, *DSE* drug side-effects, *SPM* special mention

The Kaiser-Meyer-Olkin values for general module and specific module were 0.84, and 0.75, respectively, exceeding the recommended value of 0.60, indicating a suitability of factor analysis. And Bartlett’s Tests of Sphericity were statistically significant (*P* < 0.001), also supporting the factorability of the correlation matrix. There were seven principal components (initial eigenvalues> 1) abstracted from 28 items of the general module (QLICD-PT) by factor analysis, accounting for 61.80% of the cumulative variance (Table 3). The first, third and fifth principal components mainly represented the social domain with higher loadings on GSO1 (0.65), GSO4 (0.65), GSO5 (0.71), GSO8 (0.59), GSO6 (0.58), GSO7 (0.68), GSO2 (0.77) and GSO3 (0.81). The second principal component largely reflected the psychological domain with higher loadings on GPS4 (0.74), GPS7 (0.62), GPS8 (0.60), GPS9 (0.55) and GPS11 (0.77). The fourth, sixth and seventh generally depicted the physical domain with higher loadings on GPH6 (0.86), GPH7 (0.61), GPH8 (0.85), GPH1 (0.66), GPH2 (0.75), GPH4 (0.52), GPH3 (0.74) and GPH5 (0.64). Similarly, the principal component factor analysis extracted 4 principal components from the 12 items of the specific module with the cumulative variance of 65.70%, reflecting 4 facets of this module (Table 4). The first and third components represented the two facts of respiratory symptom and drug side-effects with higher factor loadings on PT3 (0.74), PT4 (0.72), PT5 (0.64), PT6 (0.77), PT9 (0.55), PT1 (0.90) and PT2 (0.87). The second component depicted the facet of constitutional symptom with higher factor loadings on PT7 (0.83) and PT8 (0.81). The fourth component captured the facet of special mention with higher factor loadings on PT10 (0.76) and PT11 (0.85). The above analysis results confirmed the theoretical construct, showing good construct validity.

**Table 3:** Principal components and factor loadings of the general module of QLICD-PT (*n* = 200)

Items	Principal components and its variance contribution (%)						
	*P*1 (10.99)	*P*2 (10.28)	*P*3 (9.50)	*P*4 (8.78)	*P*5 (7.63)	*P*6 (7.39)	*P*7 (7.23)
GPH1	0.12	0.10	0.22	0.14	−0.03	0.66	0.07
GPH2	0.13	−0.02	0.08	0.05	0.19	0.75	0.04
GPH3	0.06	0.13	0.09	0.02	0.12	−0.03	0.74
GPH4	0.01	0.20	0.05	0.24	0.37	0.52	−0.13
GPH5	0.15	0.09	0.30	0.13	0.08	0.06	0.64
GPH6	0.25	0.07	0.08	0.86	0.22	0.14	0.03
GPH7	0.33	−0.02	0.22	0.61	− 0.19	0.24	0.30
GPH8	0.25	0.06	0.11	0.85	0.16	0.07	0.13
GPH9	0.05	0.39	0.22	0.18	−0.10	0.33	0.38
GPS1	0.56	0.08	0.14	0.33	−0.16	0.35	0.24
GPS2	0.04	0.39	0.14	0.26	0.04	0.18	0.58
GPS3	0.10	0.04	−0.23	−0.06	0.39	0.44	0.21
GPS4	0.04	0.74	0.00	−0.04	− 0.21	0.10	0.13
GPS5	0.05	0.23	0.50	0.27	0.01	−0.03	0.28
GPS6	−0.06	0.37	0.61	0.13	−0.07	0.14	−0.04
GPS7	0.17	0.62	0.46	0.05	0.10	0.07	0.07
GPS8	0.15	0.60	0.45	0.03	0.24	0.06	0.14
GPS9	0.11	0.55	0.47	0.05	0.27	−0.11	0.03
GPS10	0.74	0.05	0.24	0.11	0.07	0.17	−0.05
GPS11	0.04	0.77	0.12	0.09	0.10	0.04	0.18
GSO1	0.65	0.01	0.33	0.16	0.10	0.22	−0.02
GSO2	0.19	0.01	−0.05	0.10	0.77	0.17	0.17
GSO3	0.23	0.03	0.05	0.12	0.81	0.12	0.00
GSO4	0.65	0.18	−0.24	0.25	0.29	−0.16	0.15
GSO5	0.71	0.02	0.08	0.05	0.26	−0.05	0.08
GSO6	0.17	0.01	0.58	0.05	−0.18	0.12	0.25
GSO7	0.12	0.18	0.68	0.01	0.04	0.13	0.22
GSO8	0.59	0.19	−0.11	0.31	0.08	0.16	0.19

**Table 4:** Principal components and factor loadings of the specific module of QLICD-PT (*n* = 200)

Items	Principal components and its variance contribution (%)			
	*P*1 (21.54)	*P*2 (15.97)	*P*3 (15.13)	*P*4 (13.05)
PT1	0.18	0.16	0.90	0.07
PT2	0.22	0.22	0.87	0.02
PT3	0.74	0.11	0.31	0.33
PT4	0.72	0.13	0.21	0.20
PT5	0.64	0.05	0.13	−0.18
PT6	0.77	0.08	0.10	0.10
PT7	0.21	0.83	0.11	0.10
PT8	0.21	0.81	0.15	−0.02
PT9	0.55	0.39	−0.15	0.18
PT10	0.21	0.15	0.02	0.76
PT11	0.02	0.08	0.06	0.85
PT12	0.04	−0.53	−0.16	−0.17

Correlation coefficients among the domain scores of the QLICD-PT and SF-36 were presented in the Table 5, showing that the correlations between the same and similar domains are generally higher than those between different and non-similar domains. For example, the coefficient between the physical of QLICD-PT and physical function of SF-36 was 0.56, higher than any other coefficients in this row. Similarly, the coefficient between the social domain of QLICD-PT and social function of SF-36 was 0.47, higher than any other coefficients in this row. These confirmed the criterion-related validity to a reasonable degree and an acceptable level of the convergent and divergent validity. For Content validity, No floor effects was detected, but small ceiling effects (≤2%) were identified in the domains and in the total scale. While at the facets, significant ceiling effects were also found in three facets, ie. IND (48.5%), WIP (17.5%) and DSE (53.0%). (Table 6).

**Table 5:** Correlation coefficients among domains scores of QLICD-PT and SF-36 (*n* = 200)

QLICD-PT	SF-36							
	Physical function	Role-physical	Body pain	General health	Vitality	Social function	Role-emotional	Mental health
Physical	0.56	0.43	0.49	0.42	0.54	0.51	0.37	0.36
Psychological	0.29	0.28	0.46	0.34	0.46	0.49	0.30	0.50
Social	0.35	0.32	0.40	0.41	0.44	0.47	0.26	0.44

*All coefficients have statistical significance (*P* < 0.05)

**Table 6:** Reliability, floor and ceiling effects of the quality of life instrument QLICD-PT (*n* = 200)

Domains/facets	Internal consistency Coefficient α	Floor effects (%)	Ceiling effects (%)
Physical domain (PHD)	0.78	0.0	0.5
Basic physiologic functions (BPF)	0.50	0.0	1.0
Independence (IND)	0.83	0.5	48.5
Energy and discomfort (EAD)	0.51	1.0	3.0
Psychological domain (PSD)	0.81	0.0	0.5
Cognition (COG)	0.55	0.0	13.0
Emotion (EMO)	0.74	0.0	0.5
Will and personality (WIP)	0.20	0.0	17.5
Social domain (SOD)	0.72	0.0	2.0
Interpersonal communication (INC)	0.61	0.0	11.5
Social support and security (SSS)	0.42	0.0	4.5
Social role (SOR)	0.30	1.5	14.5
Sub-total (QLICD-GM)	0.89	0.0	0.0
Specific domain (SPD)	0.78	0.0	0.0
Respiratory symptom (RES)	0.80	0.5	1.5
Constitutional symptom (COS)	0.26	0.0	1.0
Drug side-effects (DSE)	–	2.0	53.0
Special mention (SPM)	0.62	5.5	5.5
Total (TOT)	0.90	0.0	0.0

-not acceptable/suitable

### Reliability and responsiveness

The domain-specific Cronbach’s α (for internal consistency) were higher than 0.70 for all domains. At the facet level, values of Cronbach’s α ranged from 0.20 to 0.83 (Table 6). There were statistically significant differences between before and after 2 months treatments for physical domain, specific domain, general module and the overall scale (*P* < 0.05) with SRMs ranging from 0.23 to 0.65. There were statistically significant differences between before and after 6 months treatments for all domains, general module and the overall scale (*P* < 0.05) with SRMs ranging from 0.17 to 0.72. At the domain level, significant difference was observed between 2 and 6 months treatments only for specific domain (*P* < 0.05) (Table 7).

**Table 7:** Responsiveness of the quality of life instrument QLICD-PT ($\overline{x}\pm$s) (*n* = 158)

Domains/facets	Before treatment	After 2 months treatment	After 6 months treatment	SRM (2 months)	SRM (6 months)	Variance analysis *P*	Post-hoc *P*		
							Before vs.2 months	Before vs.6 months	2 vs.6 months
Physical domain	68.79 ± 13.40	72.31 ± 13.20	74.23 ± 13.13	0.32	0.39	< 0.001	0.001	< 0.001	0.067
Basic physiologic functions	58.78 ± 13.61	62.78 ± 14.67	64.12 ± 15.08	0.23	0.33	< 0.001	0.002	< 0.001	0.283
Independence	85.55 ± 21.62	88.19 ± 19.26	89.72 ± 19.69	0.18	0.23	0.011	0.059	0.003	0.273
Energy and discomfort	63.69 ± 20.29	67.56 ± 20.74	71.20 ± 19.20	0.19	0.32	< 0.001	0.033	< 0.001	0.046
Psychological domain	67.95 ± 14.52	69.97 ± 15.02	70.66 ± 15.00	0.16	0.17	0.039	0.067	0.014	0.529
Cognition	71.12 ± 19.90	72.55 ± 19.72	75.32 ± 19.39	0.08	0.20	0.023	0.356	0.007	0.073
Emotion	65.69 ± 15.83	68.04 ± 15.86	67.86 ± 15.56	0.16	0.13	0.102			
Will and personality	72.71 ± 18.76	74.13 ± 20.45	75.79 ± 19.84	0.08	0.14	0.157			
Social domain	72.37 ± 14.83	73.80 ± 16.50	75.85 ± 14.96	0.12	0.24	0.006	0.192	0.002	0.060
Interpersonal communication	73.21 ± 16.62	74.10 ± 17.64	76.11 ± 16.40	0.06	0.16	0.097			
Social support and security	72.31 ± 17.14	74.00 ± 18.46	76.11 ± 17.57	0.12	0.22	0.017	0.203	0.004	0.112
Social role	71.20 ± 22.11	73.02 ± 23.03	75.08 ± 19.63	0.10	0.18	0.050			
Sub-total (QLICD-GM)	69.48 ± 12.55	71.81 ± 13.16	73.29 ± 12.51	0.23	0.31	< 0.001	0.010	< 0.001	0.102
Specific domain	66.07 ± 15.14	74.96 ± 14.28	77.76 ± 14.66	0.65	0.72	< 0.001	< 0.001	< 0.001	0.015
Respiratory symptom	64.69 ± 20.31	78.48 ± 18.19	81.75 ± 19.52	0.76	0.83	< 0.001	< 0.001	< 0.001	0.027
Constitutional symptom	70.25 ± 16.62	73.77 ± 13.66	74.74 ± 13.13	0.21	0.24	0.002	0.008	0.001	0.475
Drug side-effects	81.65 ± 24.83	80.85 ± 25.58	87.18 ± 21.07	0.03	0.20	0.007	0.714	0.011	0.004
Special mention	56.17 ± 26.61	63.21 ± 24.89	65.59 ± 24.30	0.28	0.35	< 0.001	< 0.001	< 0.001	0.209
Total (TOT)	68.46 ± 11.74	72.76 ± 11.58	74.63 ± 11.12	0.47	0.56	< 0.001	< 0.001	< 0.001	0.019

## Discussion

Based on the original version QLICD-GM (a generic QOL evaluation instruments for Chronic disease), this version of QLICD-GM has made several improvements to increase the comprehensibility and accessibility. For example, sexual function was adopted as one component of the facet BPF (Basic Psychological Functions) rather than listing as an independent facet since the low response rate of sex-related item among Chinese participants would influence the whole score of facet due to the privacy of sex issue in China. Moreover, several items were reworded for better straightforwardness, and similar items were combined for better simplicities. Furthermore, TB-specific domain rather than generic domain was developed and applied to measure the characteristics of pulmonary TB. By combining the modified general module QLICD-GM and the newly developed disease-specific module for pulmonary TB, we created a new QOL assessment scale QLICD-PT with psychometric strength.

Generally, a practical QOL instrument should be validated in terms of at least three aspects: validity, reliability and responsiveness [13]. In this study, correlation coefficients between the similar domains of QLICD-PT and SF-36 revealed a reasonably good criterion-related validity, and convergent and divergent validity. Correlation analysis indicated that strong association between items and their own domains/facets, but weak correlations between items and other domains/facets. Factor analysis showed that components extracted from the data coincided with the theoretical constructs of the instrument, confirming the construct validity. No floor effects and very small ceiling effects (≤2%) in the domains indicated a possibility to detect QOL improvement and deterioration over time if there are, and the item design of the scale QLICD-PT is reasonable. Internal consistency was moderate. Table 7 revealed that QOL score changes after treatment were of statistically significance on physical domain, specific domain and overall scale with SRMs ranging from 0.32 to 0.72. Given that no significant changes is expected for the psychological and social domains pertaining stable traits posttreatment, QLICD-PT can be concluded to be moderate responsiveness.

Limited efforts have been made to develop specific instruments to assess QOL of patients with tuberculosis (TB) [22], including DR-12 [23], and FACIT-TB (Functional Assessment of Chronic Illness Therapy-tuberculosis) [13]. DR-12 was developed in Indian and first published in 2003, which consists of 12 items, among them 7 cover TB symptoms and 5 relate to socio-psychological characteristics and exercise adaptation. However, response options were presented on 3 point scale, largely reducing the variation of the data collected, compromising its discriminant validity. FACIT-TB was developed in Iraq and published in 2015, which consists of 27 items of the core questionnaire, and a set of 20 items referring to diseases symptoms [13]. With more items included than DR-12, FACIT-TB is capable to pick up five domains. Being relatively brief and easy to administrate and calculate scores, FACIT-TB is in particularly suitable for the use in clinical trials [22]. However, using Consensus-Based Standards for the assortment of health status measurement instruments (COSMIN) check-list to assess methodologic quality of the HRQOL measures in development studies, Khan et al. shown that most of the studies, including DR-12 and FACIT-TB, were rated as fair to poor largely due to insufficient information collected. Therefore, it was recommended to take the advantage of good sensitivity of generic scales and excellent specificity of disease-specific scales and combine generic and disease-specific scale for a mixed scale to quantify the QOL of TB patients [24]. Our effort supports this suggestion and created a scale with both feasibility of measurement uses and qualities of methodology.

### Strengths and limitations

A recent literature failed to identify any non-English HRQOL measure developed in TB population of non-English speaking countries [22]. The current study is the first one developed in non-English language for TB patients among non-English speaking countries. The QLICD-PT is certainly subject to various limitations. The TB patients who participated in the valid study were limited with the individuals who were able to read and understand the questionnaire. Although illiterate rate in China is overall very low, however, studies have repeated demonstrated that population with low level of educational attainment and low income levels are substantially more vulnerable to TB infections, and TB prevalence is relatively high among poor communities with low education levels. Cautions must be exercised. Psychometric properties and external validity of QLID-PT should be further evaluated among population with low educational attainment when proxy interview is used. Cultural proficiency should also be carefully assessed when QLICD-PT was translated into language other than Chinese.

## Conclusions

Combining modified generic model and TB specific model, we used a rigorous method to develop a scale to better characterize QOL in Chinese patients with pulmonary TB. The 40-item QLICD-PT is a part of the QLICD instrument system, which showed acceptable certain degrees of validity, reliability, and responsiveness. Published evidence of reliability and validity indicates that FACIT-TB is the best QOL measurement tool and one of the commonly used among TB patients [22], QLICD-PT evaluated in the current study provides an alternative, at least, among Chinese.

## Abbreviations

FACIT-TB: Functional Assessment of Chronic Illness Therapy
GM: General module
HRQOL: Health-related quality of life
QLICD: Quality of Life Instruments for Chronic Diseases
QLICD-PT: Pulmonary Tuberculosis Scale of the System of Quality of Life Instruments for Chronic Diseases
QOL: Quality of life
SF-36: Medical Outcomes Study 36-Item Short-Form Health Survey
TB: Tuberculosis

## References

[1] Organization WH (2017). Global Tuberculosis Report.

[2] Silva JP, Appelberg R, Gama FM (2016). Antimicrobial peptides as novel anti-tuberculosis therapeutics. Biotechnol Adv.

[3] Huang L, Li XX, Abe EM, Xu L, Ruan Y, Cao CL, Li SZ (2017). Spatial-temporal analysis of pulmonary tuberculosis in the northeast of the Yunnan province, People's Republic of China. Infect Dis Poverty.

[4] Marra CA, Marra F, Cox VC, Palepu A, Fitzgerald JM (2004). Factors influencing quality of life in patients with active tuberculosis. Health Qual Life Outcomes.

[5] Hansel NN, Wu AW, Chang B, Diette GB (2004). Quality of life in tuberculosis: patient and provider perspectives. Qual Life Res.

[6] Macq J, Solis A, Martinez G, Martiny P, Dujardin B (2005). An exploration of the social stigma of tuberculosis in five “municipios” of Nicaragua to reflect on local interventions. Health Policy.

[7] Guo N, Marra F, Marra CA (2009). Measuring health-related quality of life in tuberculosis: a systematic review. Health Qual Life Out.

[8] Chang B, Wu AW, Hansel NN, Diette GB (2004). Quality of life in tuberculosis: a review of the English language literature. Qual Life Res.

[9] Bauer M, Leavens A, Schwartzman K (2013). A systematic review and meta-analysis of the impact of tuberculosis on health-related quality of life. Qual Life Res.

[10] Bonomi AE, Patrick DL, Bushnell DM, Martin M (2000). Validation of the United States’ version of the World Health Organization quality of life (WHOQOL) instrument. J Clin Epidemiol.

[11] European Network for Health Technolology Assessment (eunethta) (2015). Guideline Endpoints used for Relative Effectiveness Assessment: Health-Related Quality of Life and Utility Measures.

[12] Patrick DL, Deyo RA (1989). Generic and disease-specific measures in assessing health status and quality of life. Med Care.

[13] Abdulelah J, Sulaiman SAS, Hassali MA, Blebil AQ, Awaisu A, Bredle JM (2015). Development and psychometric properties of a tuberculosis-specific multidimensional health-related quality-of-life measure for patients with pulmonary tuberculosis. Value Health Reg Issues.

[14] Wan C, Tu X, Messing S, Li X, Yang Z, Zhao X, Gao L, Yang Y, Pan J, Zhou Z (2011). Development and validation of the general module of the system of quality of life instruments for chronic diseases and its comparison with SF-36. J Pain Symptom Manag.

[15] Wan C, Li H, Fan X, Yang R, Pan J, Chen W, Zhao R (2014). Development and validation of the coronary heart disease scale under the system of quality of life instruments for chronic diseases QLICD-CHD: combinations of classical test theory and generalizability theory. Health Qual Life Outcomes.

[16] Lei P, Lei G, Tian J, Zhou Z, Zhao M, Wan C (2014). Development and validation of the irritable bowel syndrome scale under the system of quality of life instruments for chronic diseases QLICD-IBS: combinations of classical test theory and generalizability theory. Int J Color Dis.

[17] Wan C, Jiang R, Tu XM, Tang W, Pan J, Yang R, Li X, Yang Z, Zhang X (2012). The hypertension scale of the system of quality of life instruments for chronic diseases, QLICD-HY: a development and validation study. Int J Nurs Stud.

[18] Ware JJ (2000). SF-36 health survey update. Spine (Phila Pa 1976).

[19] D R H (1990). Beyond internal consistency reliability: rationale and user's guide for multitrait analysis program on the microcomputer. Behav Res Methods Instrum Comp.

[20] Terwee CB, Bot SD, de Boer MR, van der Windt DA, Knol DL, Dekker J, Bouter LM, de Vet HC (2007). Quality criteria were proposed for measurement properties of health status questionnaires. J Clin Epidemiol.

[21] Husted JA, Cook RJ, Farewell VT, Gladman DD: Methods for assessing responsiveness: a critical review and recommendations. J Clin Epidemiol. 2000 53:459–468. 10.1016/s0895-4356(99)00206-1 10812317

[22] Khan S, Imtiaz A, Khan AN. Health status and quality of life in tuberculosis: systematic review of study design, instruments, measuring properties and outcomes. Health Sci J. 2017 11: 1.

[23] Dhingra VK, Rajpal S (2005). Health related quality of life (HRQL) scoring (DR-12 score) in tuberculosis--additional evaluative tool under DOTS. J Commun Dis.

[24] Brown J, Capocci S, Smith C, Morris S, Abubakar I, Lipman M (2015). Health status and quality of life in tuberculosis. Int J Infect Dis.

